# Effects of diabetes mellitus on left ventricular function and remodeling in hypertensive patients with heart failure with reduced ejection fraction: assessment with 3.0 T MRI feature tracking

**DOI:** 10.1186/s12933-022-01504-w

**Published:** 2022-05-06

**Authors:** Ge Zhang, Ke Shi, Wei-Feng Yan, Xue-Ming Li, Yuan Li, Ying-Kun Guo, Zhi-Gang Yang

**Affiliations:** 1 Department of Radiology, Functional and Molecular Imaging Key Laboratory of Sichuan Province, West China Hospital, Sichuan University, Chengdu, Sichuan China; 2 Department of Radiology, Sichuan Cancer Hospital and Institute, Sichuan Cancer Center, School of Medicine, University of Electronic Science and Technology of China, Chengdu, Sichuan China; 3 Department of Radiology, West China Second University Hospital, Sichuan University, Chengdu, Sichuan China

**Keywords:** Heart failure with reduced ejection fraction, Diabetes mellitus, Hypertension, Left ventricular dysfunction, Ventricular remodeling

## Abstract

**Background:**

Heart failure with reduced ejection fraction (HFrEF) is a major health burden worldwide with high morbidity and mortality. Comorbidities of HFrEF complicate treatment and lead to poor prognosis, among which hypertension (HTN) and diabetes mellitus (DM) are common and frequently cooccur. DM was found to have additive effects on cardiac function and structure in hypertensive patients, while its effects on the HFrEF cohort in the context of HTN remain unclear.

**Methods:**

A total of 171 patients with HFrEF were enrolled in our study, consisting of 51 HFrEF controls, 72 hypertensive HFrEF patients (HTN-HFrEF [DM−]) and 48 hypertensive HFrEF patients with comorbid DM (HTN-HFrEF [DM+]). Cardiac MRI-derived left ventricular (LV) strains, including global radial (GRPS), circumferential (GCPS) and longitudinal (GLPS) peak strain, and remodeling parameters were measured and compared among groups. The determinants of impaired LV function and LV remodeling in HFrEF patients were investigated by multivariable linear regression analyses.

**Results:**

Despite a similar LV ejection fraction, patients in the HTN-HFrEF (DM+) and HTN-HFrEF (DM−) groups showed a higher LV mass index and LV remodeling index than those in the HFrEF control group (all p < 0.05). Compared with the HTN-HFrEF (DM−) and HFrEF control groups, the HTN-HFrEF (DM+) group exhibited the most severe GLPS impairment (p < 0.001). After adjustment for covariates in HFrEF patients, DM was found to be an independent determinant of impaired LV strains in all three directions (GRPS [β = − 0.189; p = 0.011], GCPS [β = 0.217; p = 0.005], GLPS [β = 0.237; p = 0.002]). HTN was associated with impaired GLPS (β = 0.185; p = 0.016) only. However, HTN rather than DM was associated with LV remodeling in HFrEF patients in the multivariable regression analysis (p < 0.05).

**Conclusions:**

DM aggravated LV longitudinal dysfunction in hypertensive HFrEF patients without further changes in LV remodeling, indicating that HFrEF patients with comorbid HTN and DM may have a hidden high-risk phenotype of heart failure that requires more advanced and personalized management.

## Introduction

Heart failure (HF) with reduced ejection fraction (HFrEF) has become a global health concern that accounts for nearly 50% of HF cases and is attributed to an aging population and the increasing prevalence of predisposing risk factors such as hypertension (HTN) and diabetes mellitus (DM) [1, 2]. Although optimal management of HFrEF has been refined in recent decades, the disease morbidity and mortality remain high [2]. Comorbidities of HFrEF, among which HTN and DM are highly prevalent, complicate treatment and lead to poor prognosis [3].

HTN is an important risk factor for the development of HFrEF [4]. In the VICTORIA [5] and PARADIGM-HF [6] trials, a history of HTN was reported for up to 79% and 70% of patients, respectively. Once HFrEF onsets in hypertensive patients, the prognosis becomes markedly worse [7]. DM, another common risk factor for HFrEF [3], is frequently comorbid with HTN, and they share partial common pathological mechanisms of cardiac damage [8], which needs to be treated together with HTN to improve prognosis [9]. The relationship between DM and HF is bidirectional [10]; HF patients show marked insulin resistance and an increased risk of DM development, and patients with DM have a higher risk of HF and poor prognosis regardless of left ventricular ejection fraction (LVEF) [11, 12]. Therefore, among HFrEF patients, especially those with cardiovascular and noncardiovascular comorbidities, the identification of high-risk patients may be beneficial.

Previous studies have found additive effects of DM on cardiac function and structure in hypertensive patients [13–15], while its effects on the HFrEF cohort in the context of HTN have yet to be reported. Accordingly, the aim of the study was to investigate whether DM played a distinctive role in left ventricular (LV) dysfunction and remodeling in patients with HFrEF comorbid with HTN.

## Methods

### Study population

**Fig. 1:**
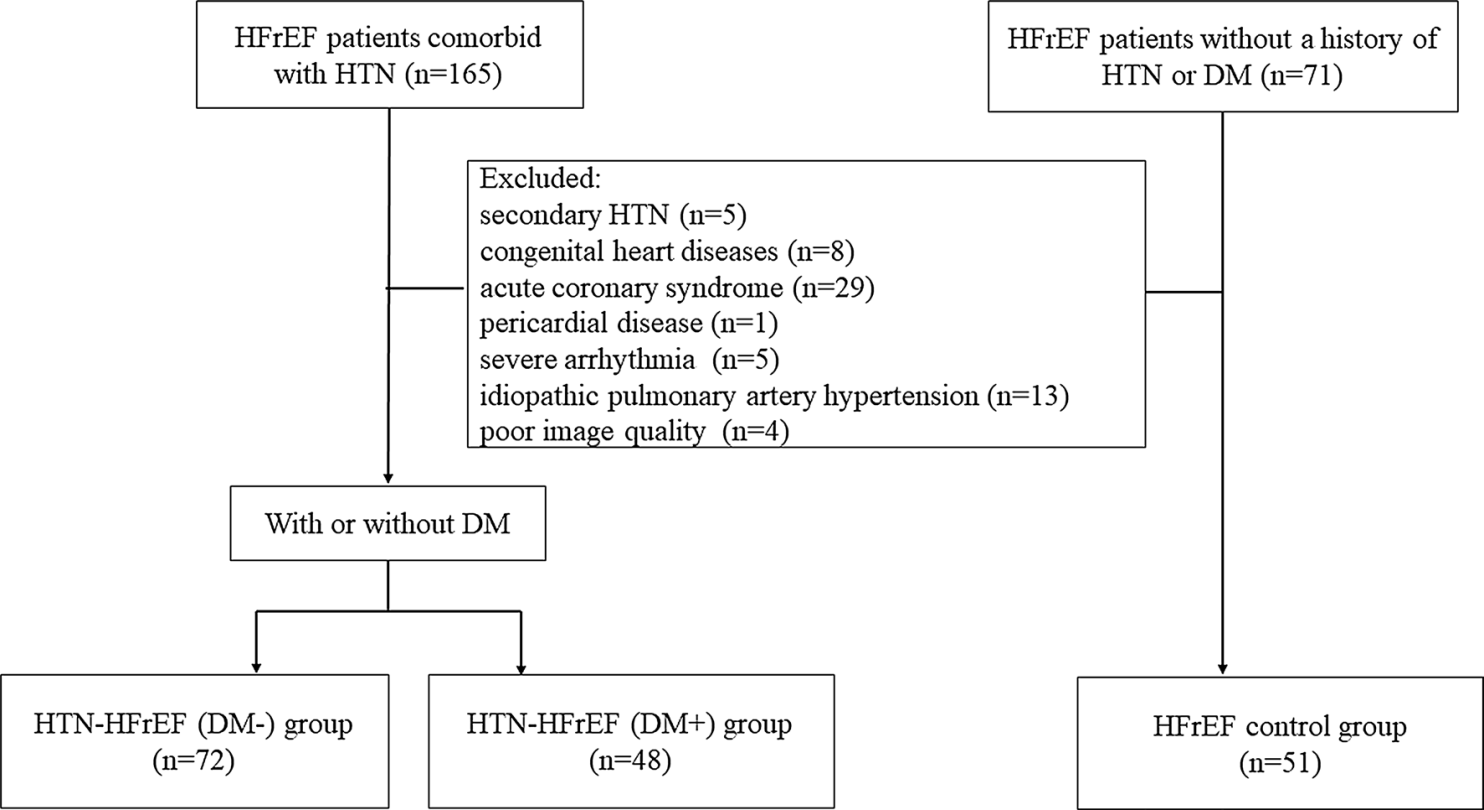
Flow diagram of the study patients. HFrEF: heart failure with reduced ejection fraction; HTN: hypertension; DM: diabetes mellitus

We retrospectively identified 120 consecutive hypertensive patients with HFrEF from October 2017 to October 2021. According to whether there was coexisting DM, patients were further divided into HTN-HFrEF (DM+) and HTN-HFrEF (DM−) groups. HFrEF was diagnosed according to the guidelines of the European Society of Cardiology (2021) [16] with symptoms and/or signs consistent with HF and LVEF ≤ 40%. All patients had a documented history of HTN (clinical systolic blood pressure [SBP] ≥ 140 mmHg and/or diastolic blood pressure [DBP] ≥ 90 mmHg) [17]. The diagnosis of DM was based on current European Society of Cardiology (2019) guidelines [18]. HFrEF patients with a history of neither HTN nor DM during the same time period were enrolled as controls. We excluded patients who had secondary HTN, congenital heart diseases, acute coronary syndrome, pericardial disease, severe arrhythmia, idiopathic pulmonary artery hypertension and cardiac MRI images with poor quality (Fig. 1). This study complied with the 1975 Declaration of Helsinki and was approved by the Biomedical Research Ethics Committee of our hospital. All patients provided written informed consent.

### Cardiac MRI protocol

All patients underwent cardiac MRI examinations on a 3.0 T MR Scanner MAGNETOM Skyra (Siemens Medical Solutions, Erlangen, Germany). Cine images, including contiguous short-axis slices covering both ventricles from the base to apex level as well as two- and four-chamber long-axis slices, were acquired by a balanced steady-state free precession (bSSPF) sequence with the following parameters: repetition time = 39.34 ms; echo time = 1.22 ms; slice thickness = 8.0 mm; flip angle = 40°; matrix = 208 × 139 pixels; and field of view = 250 × 300 mm^2^. The breath-hold technique and a standard ECG-triggering device were used to decrease motion artifacts during the entire examination process.

### Cardiac MRI postprocessing

The cine images of all patients were analyzed by the offline commercial software CVI^42^ (Circle Cardiovascular Imaging Inc., Calgary, Alberta, Canada). The LV endocardial and epicardial borders of a stack of short-axis slices were semiautomatically or manually delineated at the end-systolic and end-diastolic phases, respectively. The LV functional parameters, including LV end-diastolic volume (LVEDV), LV end-systolic volume (LVESV), LV stroke volume (LVSV), LVEF and LV mass (LVM), were computed automatically. The papillary muscles and trabeculae were excluded from the LV volume and included in the LVM. LVEDV, LVESV, LVSV and LVM were indexed to body surface area (LVEDVI, LVESVI, LVSVI and LVMI, respectively) according to the Mosteller formula [19]. The LV remodeling index, calculated as LVM/LVEDV, was also evaluated.

**Fig. 2:**
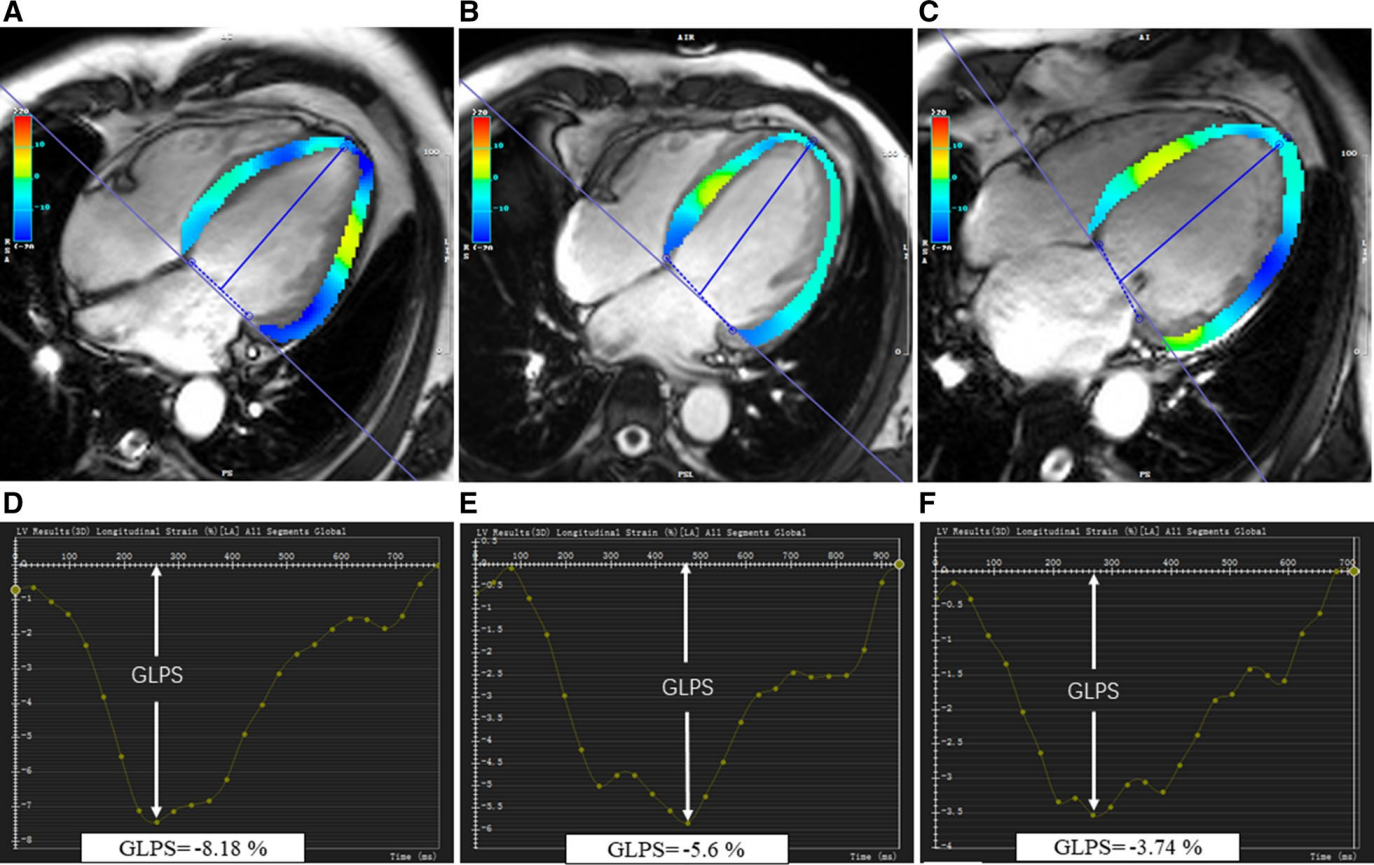
Representative cardiac MRI left ventricle pseudocolor images of long-axis four-chamber cine images at the end-systole and cardiac MRI derived global longitudinal peak strain curves of a HFrEF control patient (**A**, **D**), a hypertensive HFrEF patient (**B**, **E**), and a hypertensive HFrEF patient comorbid with diabetes mellitus (**C**, **F**)

For LV myocardial deformation analysis, LV short-axis, 2-chamber and 4-chamber long axis cine images were loaded into the feature tracking module. By delineating LV endocardial and epicardial borders at the end-diastolic phases of all cine images, LV global radial (GRPS), circumferential (GCPS) and longitudinal (GLPS) peak strain, representing relative deformation in the radial, circumferential and longitudinal directions, were automatically computed (Fig. 2).

### Reproducibility analysis

To assess intraobserver variabilities in LV global myocardial strain indices, 86 random subjects (including 62 HTN-HFrEF patients and 24 HFrEF controls) were measured by one observer (G, Z) on two separate occasions 1 month apart. A second observer (K, S) who was blinded to the imaging measurement results of the first observer and any clinical data measured the same subjects once to determine the interobserver variability.

### Statistical methods

Continuous data are presented as the mean ± standard deviation (SD) or median and interquartile range (IQR) according to the results of normality analysis. Comparisons of the baseline and cardiac MRI-derived parameters were performed by one-way analysis of variance (one-way ANOVA) followed by Bonferroni’s post-hoc test or the Kruskal–Wallis test. Categorical values are presented as numbers (percentages) and were compared by the chi-square test (or Fisher’s exact test). Univariate and multivariate linear regression analyses were used to identify the determinants of impaired LV strains and LV remodeling in the HFrEF cohort. Clinical factors and candidate variables with an absence of collinearity and a p value < 0.10 in the univariable analysis were included in the stepwise multivariable linear regression models. Intraclass correlation coefficients (ICCs) were used to evaluate intra- and interobserver reproducibility. All analyses were performed by SPSS (version 25.0, IBM SPSS Inc., Armonk, New York, USA), and a two-tailed p < 0.05 was considered indicative of significance.

## Results

### Baseline characteristics

A total of 120 hypertensive HFrEF patients, comprising 72 HTN-HFrEF (DM−) and 48 HTN-HFrEF (DM+) patients, as well as 51 HFrEF control patients, were enrolled. The main clinical characteristics of the study cohort are shown in Table 1. Compared with patients in the HFrEF control group, those in the HTN-HFrEF (DM−) and HTN-HFrEF (DM+) groups had higher body mass index (BMI) (p = 0.003), mean SBP, mean DBP and mean pulse pressure (all p < 0.001). Patients in the HTN-HFrEF (DM+) group tended to be older (p = 0.047) and had higher median amino-terminal pro-B-type natriuretic peptide (NT-proBNP) levels (p = 0.006) but a lower mean estimated glomerular filtration rate (eGFR) (p = 0.016) than those in the HFrEF control group. Sex, heart rate and HF duration were not significantly different among the three groups (all p > 0.05). As expected, both median fasting blood glucose and mean HbA1c were the highest in the HTN-HFrEF (DM+) group (all p < 0.001).

**Table 1:** Baseline characteristics of the study cohort

	HFrEF controls n = 51	HTN-HFrEF (DM−) n = 72	HTN-HFrEF (DM+) n = 48	P value
Age, yrs	50.6 ± 12.3	55.3 ± 14.8	56.9 ± 10.6	0.039
Male, n (%)	34 (66.7)	56 (77.8)	35 (72.9)	0.392
BMI, Kg/m^2^	23.3 ± 3.85	25.8 ± 4.42*	25.7 ± 4.25*	0.003
Heart rate, bpm	81.4 ± 14.5	88.1 ± 19.9	83.5 ± 14.0	0.081
SBP,mmHg	109.9 ± 17.3	132.6 ± 18.3*	130.0 ± 22.9*	< 0.001
DBP, mmHg	74.1 ± 15.2	86.4 ± 17.0*	83.8 ± 16.5*	< 0.001
PP, mmHg	35.8 ± 11.3	46.3 ± 15.4*	46.1 ± 15.7*	< 0.001
HF duration				0.081
≤ 1 yr	26 (51.0)	51 (70.8)	29 (60.4)	
> 1 and < 5 yrs	20 (39.2)	12 (16.7)	12 (25.0)	
≥ 5 yrs	5 (9.8)	9 (12.5)	7 (14.6)	
Cardiovascular risk factors, n (%)				
Smoking	20 (39.2)	31 (40.1)	24 (50.0)	0.549
Hyperlipidemia	13 (25.5)	30 (41.7)	23 (47.9)	0.057
AF	5 (9.8)	9 (12.5)	10 (20.8)	0.255
CAD	12 (23.5)	28 (38.9)	24 (50.0)*	0.023
Laboratory data				
NT-proBNP, pg/mL	1671 (647–4157)	2150 (1002–5612)	4075 (1741–8023)*	0.009
TG, mmol/L	1.28 (0.97–1.87)	1.48 (0.98–2.10)	1.66 (1.09–2.21)	0.233
TC, mmol/L	3.99 (3.19–4.88)	3.94 (3.26–4.76)	3.91 (3.39–5.01)	0.832
HDL, mmol/L	1.17 ± 0.37	1.07 ± 0.37	1.08 ± 0.28	0.226
LDL, mmol/L	2.50 ± 1.05	2.39 ± 0.93	2.43 ± 0.92	0.843
Fasting blood glucose, mmol/L	5.36 (4.71–5.89)	5.66 (5.04–6.47)	7.46 (5.77–10.00)*§	< 0.001
HbA1c (%)	5.71 ± 0.38	6.00 ± 0.54	7.43 ± 1.86*§	< 0.001
eGFR, mL/min/1.73 m^2^	82.2 ± 21.1	75.58 ± 25.6	68.1 ± 26.9	0.021
Medications, n (%)				
Beta-blocker	37 (72.5)	53 (73.6)	39 (81.3)	0.539
CCB	2 (3.9)	21 (29.2)*	10 (20.8)*	0.002
ACEI/ARB	42 (82.4)	57 (79.2)	41 (85.4)	0.681
Anti-thrombotic agents	21 (41.2)	44 (61.1)	30 (62.5)	0.047
Diuretics	48 (94.1)	65 (90.3)	39 (81.3)	0.111
Satins	17 (33.3)	35 (48.6)	26 (54.2)	0.092

Data are presented as the mean ± SD, median (Q1–Q3) or number (percentage)

HFrEF: heart failure with reduced ejection fraction; HTN: hypertension; DM: diabetes mellitus; BMI: body mass index; SBP: systolic blood pressure; DBP: diastolic blood pressure; PP: pulse pressure; HF: heart failure; AF: atrial fibrillation; CAD: coronary artery disease; NT-proBNP: amino-terminal pro-B-type natriuretic peptide; TG: triglycerides; TC: cholesterol; HDL-C: high-density lipoprotein cholesterol content; LDL-C: low-density lipoprotein cholesterol content; eGFR: estimated glomerular filtration rate; CCB: calcium-channel blocker; ACEI: angiotensin-converting enzyme inhibitor; ARB: angiotensin receptor blocker

^*^P less than 0.017 vs. HFrEF controls

^§^P less than 0.017 vs. HTN-HFrEF (DM−) group

The three groups showed a similar prevalence of smoking, hyperlipidemia and atrial fibrillation, except that coronary artery disease (CAD) was more common among patients in the HTN-HFrEF (DM+) group than in the HFrEF control group (50% vs. 23.5%, p = 0.006). There were no significant differences in the use of medications among the three groups, except for calcium channel blockers (CCBs), which were most frequently used in the HTN-HFrEF (DM−) group (p = 0.002).

### Comparison of LV functional and strain parameters

**Fig. 3:**
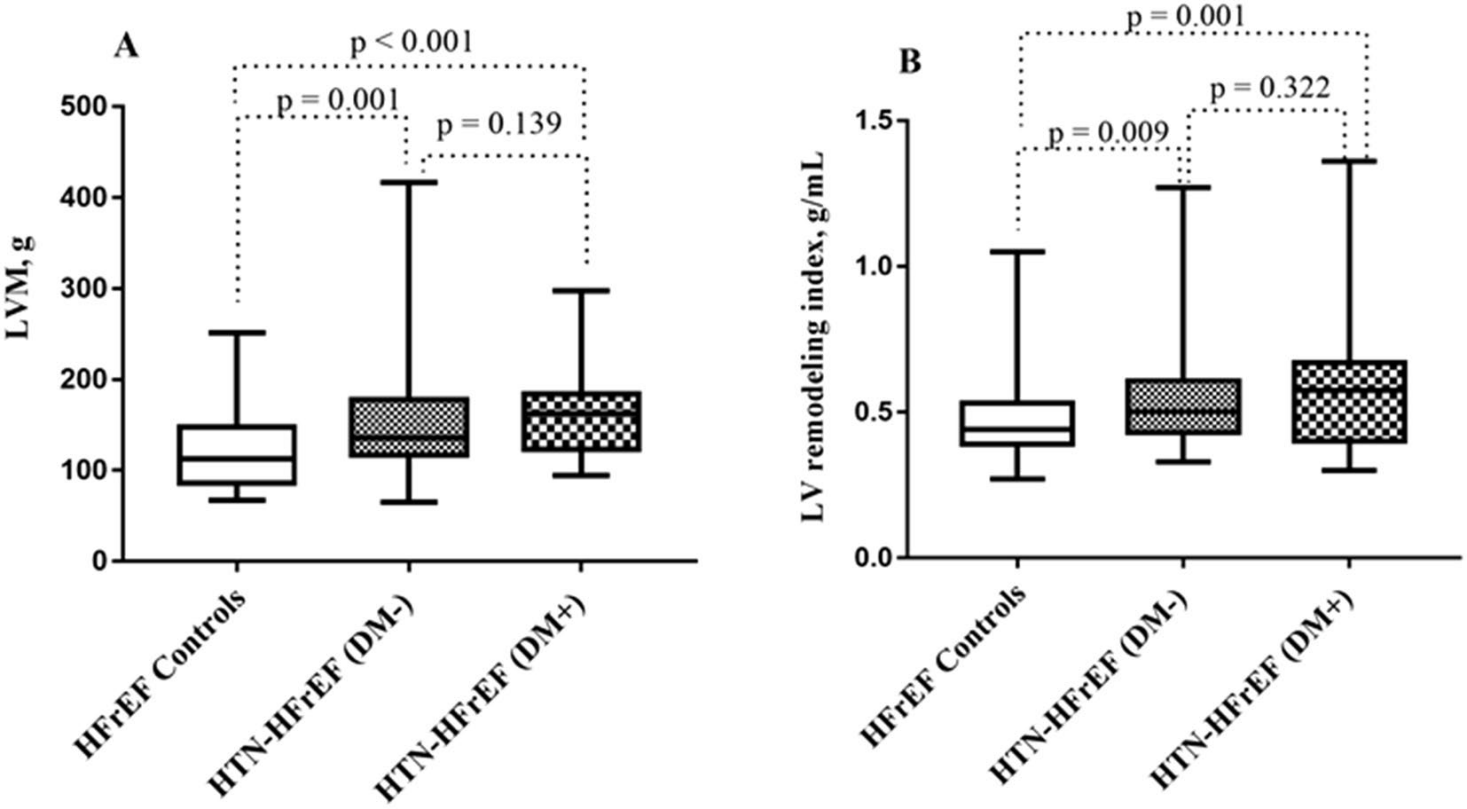
Left ventricular remodeling indices among the three groups. HFrEF: heart failure with reduced ejection fraction; HTN: hypertension; DM: diabetes mellitus; LVM: left ventricular mass

As shown in Table 2, LVEDV, LVESV, LVSV and LVEF were not significantly different among these three groups (all p > 0.05). However, patients in the HTN-HFrEF (DM−) and HTN-HFrEF (DM+) groups had a higher LVM than those in the HFrEF control group (p = 0.001 and < 0.001, respectively) (Fig. 3A), and the differences were present even when adjusted for body size (p = 0.009 and < 0.001, respectively). Moreover, similar results were found for the LV remodeling index among the three groups (HFrEF control vs. HTN-HFrEF [DM−] vs. HTN-HFrEF [DM+] group, 0.44 [IQR, 0.39–0.53] g/mL vs. 0.50 [IQR, 0.43–0.61] g/mL vs. 0.57 [IQR, 0.40–0.67] g/mL, p = 0.003) (Fig. 3B).

**Table 2:** Comparisons of cardiac MRI findings among HFrEF controls, HTN-HFrEF (DM−), and HTN-HFrEF (DM+) groups

	HFrEF controls n = 51	HTN-HFrEF (DM−) n = 72	HTN-HFrEF (DM+) n = 48	P value
Cardiac MRI parameters				
LVEDV, mL	268.80 ± 89.32	291.86 ± 106.53	293.34 ± 106.16	0.380
LVEDV index, mL/m^2^	163.75 ± 51.88	167.40 ± 62.86	165.85 ± 55.78	0.942
LVESV, mL	197.35 ± 70.00	216.66 ± 89.47	224.42 ± 92.45	0.259
LVESV index, mL/m^2^	119.95 ± 39.53	124.15 ± 52.59	127.02 ± 49.91	0.763
LVSV, mL	69.49 ± 20.67	75.19 ± 29.02	69.10 ± 27.85	0.357
LVSV index, mL/m^2^	42.57 ± 13.00	43.25 ± 17.34	39.00 ± 14.24	0.306
LVEF, %	26.54 ± 6.71	26.71 ± 7.81	24.78 ± 8.34	0.362
LVM, g	112.22 (86.02–147.46)	135.48 (116.55–177.56)*	161.92 (122.82–184.00)*	< 0.001
LVM index, g/m^2^	70.51 (56.39–82.13)	76.60 (67.71–94.04)*	90.54 (73.84–103.78)*	< 0.001
LV remodeling index, g/mL	0.44 (0.39–0.53)	0.50 (0.43–0.61)*	0.57 (0.40–0.67)*	0.003
Myocardial strain parameters				
GRPS, %	10.22 ± 4.64	9.01 ± 4.70	7.53 ± 3.31*	0.010
GCPS, %	− 8.55 ± 2.80	− 7.85 ± 3.23	− 6.73 ± 2.78*	0.011
GLPS, %	− 6.68 ± 2.03	− 5.39 ± 2.29*	− 4.02 ± 1.43*§	< 0.001

Data are presented as the mean ± SD, or media (Q1–Q3)

HFrEF: heart failure with reduced ejection fraction; HTN: hypertension; DM: diabetes mellitus; LVEDV: left ventricular end-diastolic volume; LVESV: left ventricular end-systolic volume;

LVSV: left ventricular stroke volume; LVEF: left ventricular ejection fraction; LVM: left ventricular mass; GRPS: global radial peak strain; GCPS: global circumferential peak strain; GLPS: global longitudinal peak strain

^*^P less than 0.017 vs. HFrEF controls

^§^P less than 0.017 vs. HTN-HFrEF (DM−) group

**Fig. 4:**
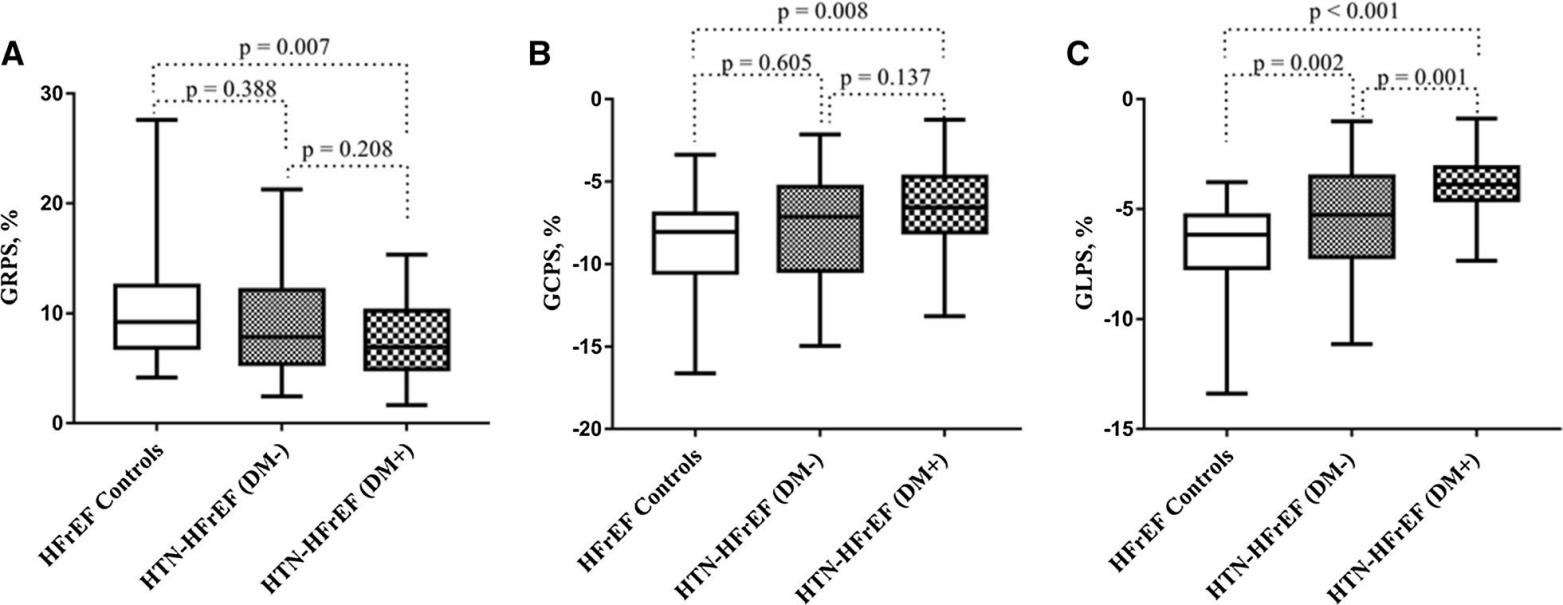
Global left ventricular strain indices among the three groups. HFrEF: heart failure with reduced ejection fraction; HTN: hypertension; DM: diabetes mellitus; GRPS: global radial peak strain; GCPS: global circumferential peak strain; GLPS: global longitudinal peak strain

The LV GLPS declined progressively from the HFrEF control group to the HTN-HFrEF (DM−) group to the HTN-HFrEF (DM+) group (− 6.68 ± 2.03 vs. − 5.39 ± 2.29 vs. − 4.02 ± 1.43, p < 0.001). Compared with those in the HFrEF control group, smaller LV GRPS and LV GCPS were observed in the HTN-HFrEF (DM+) group (p = 0.007 and = 0.008, respectively) but preserved in the HTN-HFrEF (DM−) group (both p > 0.05) (Fig. 4).

### Determinants of LV dysfunction and remodeling

After multivariable adjustment for covariates among all HFrEF patients, DM was found to be an independent determinant of impaired GRPS (β = − 0.189; p = 0.011), GCPS (β = 0.217; p = 0.005) and GLPS (β = 0.237; p = 0.002) (Table 3). HTN (β = 0.185; p = 0.016), NT-proBNP level (β = 0.200; p = 0.004) and LVM (β = 0.214; p = 0.003) were associated only with impaired GLPS. Moreover, obesity was associated with impaired GRPS (β = − 0.227; p = 0.002) and GCPS (β = 0.214; p = 0.004). eGFR was associated only with impaired GCPS (β = 0.178; p = 0.020).

**Table 3:** Determinants of LV dysfunction in patients with HfrEF

	GRPS				GCPS				GLPS			
	Univariable		Multivariable		Univariable		Multivariable		Univariable		Multivariable	
	β	P value	β	P value	β	P value	β	P value	β	P value	β	P value
Age^#^, yrs	0.092	0.229			− 0.104	0.175			0.007	0.930		
Male	− 0.118	0.124			0.098	0.202			0.168	0.028		
Obesity^&^	− 0.237	0.002	− 0.227	0.002	0.251	0.001	0.214	0.004	0.165	0.031		
PP^#^, mmHg	0.140	0.067			− 0.162	0.035			− 0.023	0.763		
HTN	− 0.187	0.015			0.173	0.024			0.378	< 0.001	0.185	0.016
DM	− 0.201	0.008	− 0.189	0.011	0.208	0.006	0.217	0.005	0.384	< 0.001	0.237	0.002
CAD	− 0.078	0.312			0.012	0.877			0.085	0.268		
NT-proBNP*, pg/mL	− 0.062	0.420			0.042	0.587			0.307	< 0.001	0.200	0.004
eGFR, mL/min/1.73 m2	− 0.069	0.380			0.141	0.071	0.178	0.020	− 0.151	0.054		
LVM, g	− 0.199	0.009			0.165	0.031			0.335	< 0.001	0.214	0.003

β is the adjusted regression coefficient

LV: left ventricular; HFrEF: heart failure with reduced ejection fraction; GRPS: global radial peak strain; GCPS: global circumferential peak strain; GLPS: global longitudinal peak strain; PP: pulse pressure; HTN: hypertension; DM: diabetes mellitus; CAD: coronary artery disease; NT-proBNP: amino-terminal pro-B-type natriuretic peptide; eGFR: estimated glomerular filtration rate; LVM: left ventricular mass

^#^Changes in dependent variables per 10-unit increases

^&^Subjects with body mass index ≥ 25 kg/m^2^ were classified into the obese group, as proposed by the World Health Organization for Asian populations

^*^NT-proBNP was log-transformed before being included in the regression analysis

The univariable analysis showed that HTN, DM and eGFR were common variables associated with an increased LVM and LV remodeling index (all p < 0.05) (Table 4). After multivariable adjustment, HTN remained an independent determinant of increased LVM (β = 0.253, p < 0.001) and LV remodeling index (β = 0.219, p = 0.005) among all HFrEF patients.

**Table 4:** Determinants of LV remodeling in patients with HfrEF

	LVM				LV remodeling index			
	Univariable		Multivariable		Univariable		Multivariable	
	β	P value	β	P value	β	P value	β	P value
Age^#^, yrs	− 0.170	0.026	− 0.271	< 0.001	− 0.017	0.826		
Male	0.368	< 0.001	0.293	< 0.001	0.115	0.135		
Obesity^&^	0.309	< 0.001	0.145	0.039	0.085	0.267		
HTN	0.281	< 0.001	0.253	< 0.001	0.231	0.002	0.219	0.005
DM	0.167	0.029			0.215	0.005		
NT-proBNP*, pg/mL	0.140	0.068			0.067	0.382		
eGFR, mL/min/1.73 m^2^	− 0.230	0.003	− 0.262	< 0.001	− 0.195	0.012	− 0.156	0.043

β is the adjusted regression coefficient

^#^Changes in dependent variables per 10-unit increase

^&^Subjects with body mass index ≥ 25 kg/m^2^ were classified into the obese group, as proposed by the World Health Organization for Asian populations

^*^NT-proBNP was log-transformed before being included in the regression analysis

Abbreviations as listed in Table 3

### Intra- and interobserver variability

There was excellent intraobserver agreement in terms of GRPS (ICC = 0.949, 95% CI 0.897–0.975), GCPS (ICC = 0.975, 95% CI 0.949–0.987) and GLPS (ICC = 0.955, 95% CI 0.910–0.978). Similarly, the interobserver agreement in terms of GRPS (ICC = 0.941, 95% CI 0.883–0.971), GCPS (ICC = 0.952, 95% CI 0.904–0.976) and GLPS (ICC = 0.942, 95% CI 0.885–0.971) was excellent.

## Discussion

The study investigated the combined effects of DM on cardiac MRI-derived LV function and remodeling in hypertensive patients with HFrEF and explored the independent predictors of LV dysfunction and remodeling. The main findings were as follows: (1) compared with the HFrEF control cohort, HTN impaired LV GLPS, but preserved LV GRPS and GCPS, whereas DM deteriorated LV strains in all three directions (radial, circumferential, and longitudinal). Moreover, DM deteriorated GLPS in patients with HFrEF in the context of HTN. (2) For HFrEF patients, HTN was significantly associated with impaired GLPS. DM was found to be an independent determinant of impaired LV strains in all three directions. (3) Patients with HFrEF comorbid with DM and HTN displayed an increased LVM and LV remodeling index, in which HTN plays the predominant role. Our study indicated that DM aggravated LV longitudinal dysfunction without causing notable changes in LV geometry in HFrEF patients in the context of HTN; therefore, HFrEF patients with comorbid DM and HTN may have a hidden high-risk phenotype of HFrEF that needs more advanced and personalized management.

### DM aggravates LV longitudinal dysfunction in patients with HFrEF comorbid with HTN

HTN, the most common comorbidity and a major risk factor for HFrEF, can lead to diffuse interstitial fibrosis and LV hypertrophy [20]. Subendocardial fibers are most vulnerable to the adverse effects of interstitial fibrosis [21] and coronary microvascular dysfunction secondary to LV hypertrophy [22], so longitudinal dysfunction representing as GLPS can appear at an earlier stage. Our study showed that in patients with HFrEF comorbid with HTN, GLPS was impaired with preserved GRPS and GCPS. In contrast, DM impaired LV deformation in all three directions when comorbid with HFrEF. Previous studies reported consistent results that DM reduced LV strains in patients with or without HF [23–25]. A meta-analysis [26] validated a high prevalence of LV systolic dysfunction among HFrEF patients with comorbid DM. The underlying mechanisms of how DM affects LV function are not fully understood. This may be due to the synthesis effect of metabolic disorder, excitation–contraction coupling impairment, microvasculature dysfunction and extracellular matrix fibrosis [14, 27].

Despite the similar LVEF values in the HTN-HFrEF (DM−) and HTN-HFrEF (DM+) groups, our study demonstrated that DM further impaired LV GLPS in HFrEF patients with comorbid HTN. To our knowledge, this was the first study investigating the effects of DM on LV function in a hypertensive HFrEF cohort. Insulin resistance increases oxidative stress, facilitating cardiomyocyte damage, interstitial fibrosis and LV hypertrophy [28], thereby aggravating the impairment of subendocardial fibers, which may partly explain the additive effect. The underlying comprehensive factors need to be studied further. Studies have shown that DM impaired GLPS in patients without HF to heart failure with preserved ejection fraction patients to HFrEF patients [14, 25, 29], indicating that the adverse effects caused by DM may be more extensive. Evidence has shown that GLPS is an independent predictor for adverse outcomes in patients with HFrEF [30]; thus, impaired GLPS in hypertensive HFrEF patients caused by comorbid DM may help identify high-risk patients.

### Determinants of impaired LV function in HFrEF patients

In this study, we conducted multivariable linear regression analysis to identify determinants of impaired LV function in HFrEF patients for the first time. As two common risk factors for HFrEF, HTN and DM share some common pathological mechanisms leading to cardiac damage [8], and our study showed that both HTN and DM were associated with impaired LV function, which may partly explain the worse outcomes when HFrEF patients had these two comorbidities. Obesity is a risk factor for the incidence of overall HF and can lead to changes in LV structure and function [31]. In our study, obesity was also found to be an independent variable related to impaired LV function, which was consistent with the study by Wang et al. [32]. Similar to DM [14], obesity was reported to cause functional microvascular impairment [33], which in turn may cause LV dysfunction. Moreover, our study found that eGFR was associated with impaired LV function, which may be caused by volume overload. Impairment of renal function is common in patients with HFrEF and is associated with worse outcomes. The use of sodium-glucose cotransporter 2 inhibitors (e.g., dapagliflozin) can help slow the rate of decline in eGFR in patients with HFrEF and improve prognosis [34].

Studies have found that the NT-proBNP concentration has diagnostic and prognostic relevance [35]. Recent clinical trials have demonstrated that the NT-proBNP concentration after treatment is correlated with reversed cardiac remodeling and function [36] and better informs prognosis [37]. Our study showed that the NT-proBNP level was independently associated with impaired GLPS but not with GCPS or GRPS. This finding highlights the prognostic value of GLPS for HFrEF patients. Elevated LVM was reported to be an independent predictor of cardiovascular death and HF [38]. Notably, LVM was also found to be related to GLPS in our study. Both HTN and DM lead to increased LVM and LV hypertrophy associated with interstitial fibrosis. Interstitial fibrosis may be responsible for increased LV stiffness and reduced end-diastolic muscle fiber length, which therefore results in reduced myocardial systolic strain [39].

### The effects of HTN and DM on LV remodeling in HFrEF patients

Compared with HFrEF controls, HFrEF patients with concomitant HTN and DM displayed an increased LVM and LV remodeling index, which was also the case for patients without HF, as shown in a study by Li et al. [14]. Both HTN and DM are characterized by hypertrophy, which is a growth in LVM caused by increased cardiomyocyte size; however, in the multivariable regression analysis, HTN rather than DM serves as an independent determinant of LV remodeling. Since the effects of concomitant cardiovascular risk factors, such as HTN, obesity and DM, on LV remodeling are not additive but synergistic [40], our results suggested that HTN plays the predominant role in LV remodeling in HFrEF patients. More importantly, similar LVEF and LV geometries observed between the hypertensive HFrEF subgroups may mask the impaired GLPS caused by comorbid DM in hypertensive HFrEF patients in a clinical setting, which may delay treatment and lead to poor outcomes. Therefore, early intervention in HFrEF patients with comorbid HTN and DM may provide potential benefits. The results also indicated that there may be pathways other than LV remodeling that result in additive effects of DM on LV longitudinal function in hypertensive HFrEF patients. The underlying mechanisms need further investigation.

## Limitations

Our study has several limitations. First, the study found that DM deteriorated GLPS in hypertensive HFrEF patients. Due to the limitation of this cross-sectional study, the prognosis caused by impaired GLPS in hypertensive HFrEF patients with comorbid DM was not investigated in our study. Second, this was a single-center study, and future multicenter studies with a larger number of patients are needed to validate our findings. Third, HFrEF patients usually have several cardiovascular risk factors, including obesity and coronary heart disease, which may have potential adverse effects on LV function. However, we included several common risk factors in the multivariable regression analysis and found that DM was still an independent determinant of LV function. It would be interesting to understand how DM affects LV function and the HF phenotype alone in the future, for example, by performing animal studies. Fourth, we didn’t assess the etiology of HFrEF of each patient, future studies could be investigated with larger cohort to evaluate the effects of different etiologies on LV strains.

## Conclusions

Both HTN and DM had adverse effects on LV function in patients with HFrEF. In HFrEF patients with comorbid HTN and DM, DM was found to aggravate LV longitudinal dysfunction in the absence of further changes in LV remodeling, an occult injury that was difficult to identify in the clinical setting. Early identification and initiation of phenotype-specific treatment of these high-risk patients may improve prognosis.

## Data Availability

The datasets used and analyzed during the current study are available from the corresponding author on reasonable request.

## Abbreviations

HF: Heart failure
HFrEF: Heart failure with reduced ejection fraction
HTN: Hypertension
DM: Diabetes mellitus
LV: Left ventricular
LVEF: Left ventricular ejection fraction
MRI: Magnetic resonance imaging
LVEDV: Left ventricular end-diastolic volume
LVESV: Left ventricular end-systolic volume
LVSV: Left ventricular stroke volume
LVM: Left ventricular mass
GRPS: Global radial peak strain
GCPS: Global circumferential peak strain
GLPS: Global longitudinal peak strain
IQR: Inter quartile range
BMI: Body mass index
NT-proBNP: Amino-terminal pro-B-type natriuretic peptide
CAD: Coronary artery disease
